# *Tau* Knockout and α-*Synuclein A53T* Synergy Modulated Parvalbumin-Positive Neurons Degeneration Staging in Substantia Nigra Pars Reticulata of Parkinson’s Disease-Liked Model

**DOI:** 10.3389/fnagi.2021.784665

**Published:** 2022-01-11

**Authors:** Meige Zheng, Yanchang Liu, Zhaoming Xiao, Luyan Jiao, Xian Lin

**Affiliations:** ^1^Department of Orthopaedics, The Second Hospital of Anhui Medical University, Hefei, China; ^2^Nuwacell Biotechnologies Co., Ltd, Hefei, China; ^3^Guangdong Province Key Laboratory of Brain Function and Disease, Zhongshan School of Medicine, Sun Yat-sen University, Guangzhou, China; ^4^Department of Anatomy, Zhongshan School of Medicine, Sun Yat-sen University, Guangzhou, China

**Keywords:** *Tau* knockout, α-*synuclein*, parvalbumin, NeuN, degeneration staging

## Abstract

The loss of parvalbumin-positive (PV^+^) neurons in the substantia nigra pars reticulata (SNR) was observed in patients with end-stage Parkinson’s disease (PD) and our previously constructed old-aged *Pitx3-A53T*α*-Syn* × *Tau^–/–^* triple transgenic mice model of PD. The aim of this study was to examine the progress of PV^+^ neurons loss. We demonstrated that, as compared with non-transgenic (*nTg*) mice, the accumulation of α-*synuclein* in the SNR of aged *Pitx3-A53T*α*-Syn* × *Tau^–/–^* mice was increased obviously, which was accompanied by the considerable degeneration of PV^+^ neurons and the massive generation of apoptotic NeuN^+^TUNEL^+^ co-staining neurons. Interestingly, PV was not costained with TUNEL, a marker of apoptosis. PV^+^ neurons in the SNR may undergo a transitional stage from decreased expression of PV to increased expression of NeuN and then to TUNEL expression. In addition, the degeneration of PV^+^ neurons and the expression of NeuN were rarely observed in the SNR of *nTg* and the other triple transgenic mice. Hence, we propose that *Tau* knockout and α*-syn A53T* synergy modulate PV^+^ neurons degeneration staging in the SNR of aged PD-liked mice model, and NeuN may be suited for an indicator that suggests degeneration of SNR PV^+^ neurons. However, the molecular mechanism needs to be further investigated.

## Introduction

The most predominant pathological feature of Parkinson’s disease (PD) is the massive loss of dopaminergic neurons in the substantial nigra pars compacta (SNC) (Poewe et al., 2017). It may be closely related to the massive accumulation of α-*synuclein* (α-syn) and the aberrant expression of the microtubule-associated protein tau (Bassil et al., 2021; Han et al., 2021), both of which have been identified as the first two genes of the population attributable risk underlying PD in genome-wide association studies (Simon-Sanchez et al., 2009).

The majority of PD research has focused on the SNC, while substantia nigra pars reticulata (SNR), as the other part of the substantia nigra, has received little attention (Dionísio et al., 2021; Pirooznia et al., 2021). Previous studies showed that the activity of parvalbumin-positive (PV^+^) neurons in the SNR were affected in PD animal models (Wichmann et al., 1999; Mallet et al., 2019; Pamukcu et al., 2020). The loss of parvalbumin (PV) immunoreactivity was detected even in patients with end-stage PD (Hardman et al., 1996). Recently, Lilascharoen et al. (2020) indicated that the frequency of quantal-like inhibitory postsynaptic currents was significantly decreased in SNR neurons, which might indeed contribute to immobility in the 6-hydroxydopamine-lesioned PD mice model, as selective manipulation of the SNR-projecting external globus pallidus PV^+^ neurons alleviated the locomotor deficit. Our previous study found that the *Pitx3-A53T*α*-Syn* × *Tau^–/–^* triple transgenic mice model of PD showed anxiety-like behavior among 12- to 18-mouth-old, which may be related to the substantial degeneration of PV^+^ neurons in the SNR (Jiao et al., 2020). However, the progress and stage marker of PV^+^ neurons loss are still unclear.

In this study, we demonstrated that, as compared with non-transgenic (*nTg*) mice, the accumulation of α-syn in the SNR of aged *Pitx3-A53T*α*-Syn* × *Tau^–/–^* mice was increased obviously, which was accompanied by the considerable loss of PV^+^ neurons and the massive generation of apoptotic NeuN^+^TUNEL^+^ neurons. PV^+^ neurons in the SNR may undergo a transitional stage from decreased PV expression to increased NeuN expression and then to TUNEL expression of apoptosis, whereas no co-localization was observed for PV and TUNEL during the progress.

## Materials and Methods

### Construction of Triple Transgenic Mice

As described previously (Jiao et al., 2020), the line of pituitary homeobox 3 (*Pitx3*) promoter-controlled tetracycline transactivator (*tTA*) (*Pitx3-tTA*) mice, the lines of human α-syn A53T inducible transgenic mice (*tetO-A53T*) and human wild-type Tau inducible transgenic mice (*tetO-hTau*), in which the expression of human α-syn *A53T* and human wild-type Tau were under the transcriptional control of tetracycline operator (*tetO*), and the line of *Tau^–/–^* mice were used to generate triple transgenic mice and maintained on C57BL/6J background. The mice were reared under specific-pathogen-free (SPF) conditions, with 12-h light/12-h dark cycles and fed a regular diet *ad libitum*. All experimental procedures performed in this study were approved by the Institutional Animal Care and Use Committee of Sun Yat-sen University.

### Genotype Identification

The genotype of the mice was determined by PCR analysis of genomic DNA extracted from tail biopsy and had been further validated histologically, as described in the previous study (Jiao et al., 2020).

### Immunofluorescence

The mice were perfused transcardially with phosphate-buffered saline (PBS). Brains were separated and soaked in 4% paraformaldehyde for 48 h. Then, 30% sucrose solution was used for dehydration treatment. A cryostat (Leica SM 2010R, Germany) was used to cut the brains into continuous slices with a thickness of 40 μm. The following primary and secondary antibodies were used as recommended by the manufacturers: rabbit anti-human/mouse α-*synuclein* (α-syn; Santa Cruz Biotechnology, United States, sc-7011-R, 1:1000), mouse anti-PV (Sigma-Aldrich, United States, P3088, 1:500), rabbit anti-PV (Abcam, United States, ab11427, 1:1000), rabbit anti-c-fos (Sigma-Aldrich, United States, F7799, 1:1000), mouse anti-neuronal nucleus (NeuN; Chemicon, United States, MAB 377, 1:500), and Alexa 488 or Alexa 555 conjugated secondary antibody (Invitrogen, United States, 1:500). For antibodies produced in mice, the mouse-on-mouse immunodetection kit (Vector Laboratories, United States, BMK-2202) was used following the manufacturer’s protocol. A laser scanning confocal microscope (LSM 710; Zeiss, Germany) was used for observing and photographing. The paired images in all the figures were acquired under the same conditions and processed uniformly after collection.

### Terminal Deoxynucleotidyl Transferase-Mediated dUTP Nick End Labeling Staining

According to the manufacturer’s protocol, apoptotic cells were labeled with an apoptosis detection kit (Roche, United States, 11684795910). The frozen sections were adhered to the slides and air-dried at 50°C for 30 minutes. Slides were rinsed in PBS for 5 min × 4 times, and then immersed in the permeabilization solution (0.1% sodium citrate, containing0.1% TritonX-100) and incubated on ice (2–8°C) for 2 min. Slides were washed in PBS for 5 min × 4 times. Afterward, the terminal deoxynucleotidyl transferase-mediated dUTP nick end labeling (TUNEL) reaction mixture was added to the slides and incubated at 37°C in the dark for 1 h. Slides were then rinsed in PBS for 5 min × 4 times and in ddH2O for 5 min × 3 times. After air-drying for 2 h at 37°C in the dark, the slides were mounted with ProLong^®^ Gold anti-fading reagent (Invitrogen, United States, 1724814) before analysis.

### Image Analysis

Cell counting of TUNEL^+^, NeuN^+^, and PV^+^ cells was performed in serial coronal sections across the SNR (every fourth from bregma, −3.28 to −4.04 mm). Fluorescence images of TUNEL, NeuN, and PV on each of the coronal sections in the SNR of 2-, 6-, and 12-month-old *Pitx3-A53T*α*-Syn* × *Tau^–/–^* mice were captured with a laser-scanning confocal microscope (LSM 710; Zeiss, Germany) at 20 × magnification. The numbers of TUNEL^+^, NeuN^+^, and PV^+^ cells, PV and NeuN double-positive cells, and TUNEL and NeuN double-positive cells were counted with NIS-Elements BR Imaging software (Nikon Instruments, Japan). The percentage of PV and NeuN double-positive cells to the total number of PV^+^ cells and the percentage of TUNEL and NeuN double-positive cells to the total number of TUNEL^+^ or NeuN^+^ cells in the SNR were determined.

To quantify the relative immunofluorescence intensity of α-syn, c-fos, and PV in the SNR of mice, images were taken using identical settings and then analyzed using ImageJ software (NIH, United States). To measure c-fos and PV fluorescent intensity, only fluorescence within the PV^+^/c-fos^+^ co-staining neurons in the SNR was analyzed. All the images were converted to an 8-bit color scale and the background was subtracted. Areas of interest were selected by the freehand selection tools and subjected to measurement by mean gray value to determine the average intensity.

Three mice were used per genotype and at each time point. Counters were blinded to the information of the samples.

### Statistics

GraphPad Prism 7 (GraphPad Software, United States) was used for statistical analysis. Data were expressed as mean ± SEM. The one-way analysis of variance was used to compare the means of different groups, followed by Tukey’s honestly significant difference *post hoc* test, and significance was set at *P* < 0.05.

## Results

### *Tau* Knockout Exacerbated the Accumulation of α-Syn in the Substantia Nigra Pars Reticulata of α*-Syn A53T* Conditional Transgenic Mice

As described in our previous study, we continued to use the triple transgenic mice that overexpressed PD-related α*-syn A53T* missense mutation in the midbrain dopaminergic neurons with different tau gene dosage from high to low, which were named as *Pitx3-A53T*α*-Syn* × *hTau*, *Pitx3-A53T*α*-Syn* × *Tau*^+^/^+^, *Pitx3-A53T*α*-Syn* × *Tau*^+/–^, and *Pitx3-A53T*α*-Syn* × *Tau^–/–^* mice, respectively (Jiao et al., 2020).

**FIGURE 1:**
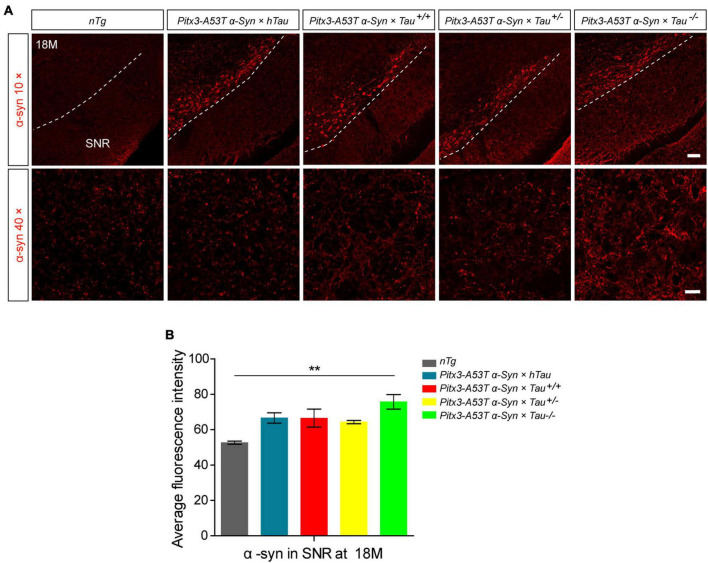
α-syn accumulation was increased in the substantia nigra pars reticulata (SNR) of 18-month-old *Pitx3-A53T*α*-Syn* × *Tau^–/–^* mice. **(A)** α-syn immunostaining in the SNR of 18-month-old mice. compared to *nTg* mice, α-syn was obviously overexpressed in dopaminergic neurons in SNC of the triple transgenic mice. White dotted lines demarcate the boundary between SNC and SNR. The ventrolateral area was considered as SNR. Scale bars: low magnification (10×), 100 μm; higher magnification (40×) in SNR, 25 μm. **(B)** Quantitative analysis of α-syn fluorescence intensity in SNR of 18-month-old mice. *n* = 3 per genotype. Values are mean ± SEM. **P < 0.01 (*Pitx3-A53T*α*-Syn* × *Tau^–/–^* vs *nTg*).

Considering that *Pitx3-A53T*α*-Syn* × *Tau^–/–^* mice specifically developed severe degeneration of PV^+^ neurons in the SNR at 18-month-old, we presumed that this may be related to the accumulation of α-syn. We stained α-syn in the SNR of 18-month-old mice. The results showed that compared with *nTg* mice, α-syn was significantly overexpressed in the SNC of all triple transgenic mice, as expected (Figure 1A). But more prominently, *Pitx3-A53T*α*-Syn* × *Tau^–/–^* mice had the highest level of α-syn (^**^P < 0.01, vs. *nTg*), while other triple transgenic mice had almost the same α-syn content in the SNR with no statistical difference as compared to *nTg* mice (Figures 1A,B). These results indicated that tau knockout could modestly exacerbate the accumulation of α-syn in the SNR of α*-syn A53T* conditional transgenic mice at old age.

### *Pitx3-A53T*α*-Syn* × *Tau^–/–^* Mice Showed Actually Decreased Parvalbumin-Positive and c-fos^+^ Neurons in the Substantia Nigra Pars Reticulata at 18-Month-Old

**FIGURE 2:**
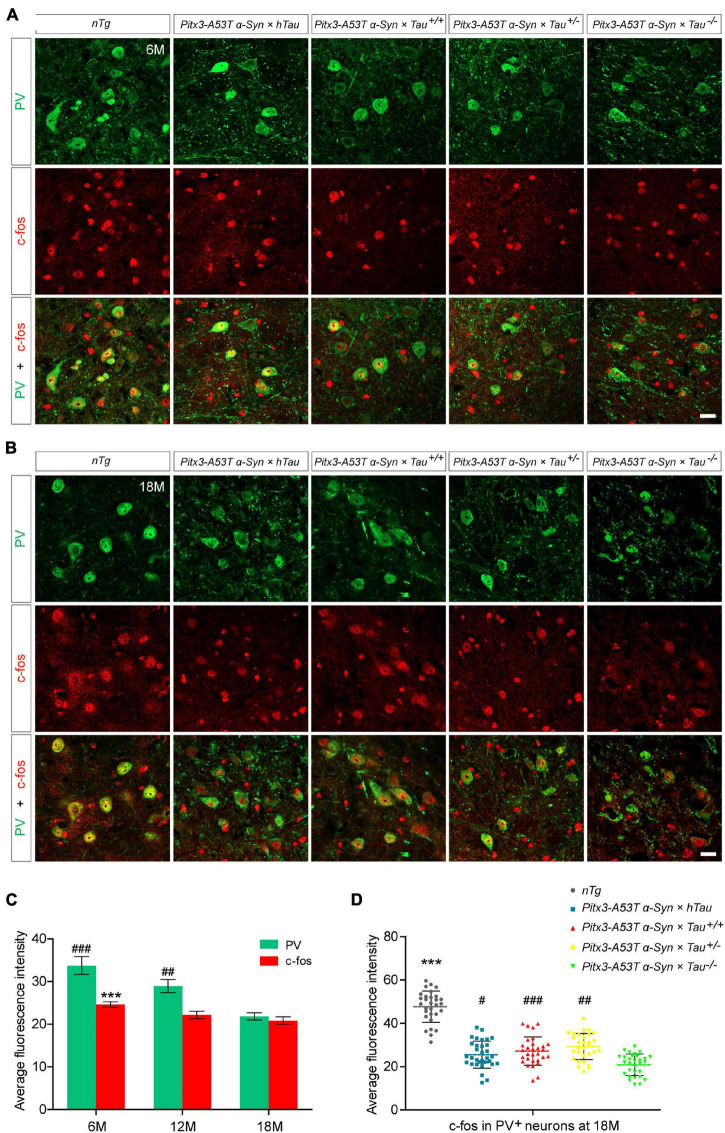
Decreased expression of c-fos in the parvalbumin-positive (PV^+^) neurons in the SNR of α*-syn A53T* transgenic mice. **(A,B)** PV (green) and c-fos (red) costained in the SNR of 6- **(A)** and 18-month-old **(B)** mice. Scale bars: 25 μm. **(C)** Quantification of c-fos and PV fluorescence mean intensity in PV^+^/c-fos^+^ co-staining cells in the SNR of 6-, 12- and 18-month-old *Pitx3-A53T*α*-Syn* × *Tau^–/–^* mice (*n* = 30 cells for each sample, 3 mice for each time point). Values are mean ± SEM. ***P < 0.001 (c-fos, 6-month-old vs 18-month-old *Pitx3-A53T*α*-Syn* × *Tau^–/–^*); ^##^*P* < 0.01, ^###^*P* < 0.001 (PV, 6- and 12-month-old vs 18-month-old *Pitx3-A53T*α*-Syn* × *Tau^–/–^*). **(D)** Representative dot plots of c-fos fluorescence mean intensity in PV^+^ neurons in the SNR of 18-month-old mice (*n* ≥ 30 cells for each sample, 3 mice per genotype). ***P < 0.001 (*nTg* vs triple transgenic); ^#^*P* < 0.05, ^##^*P* < 0.01, ^###^*P* < 0.001 (other triple transgenic vs *Pitx3-A53T*α*-Syn* × *Tau^–/–^*).

As the expression product of the Fos gene, c-fos is most commonly used to detect the activity of neurons in the central nervous system. The expression level of c-fos is directly proportional to the activity of neurons (Dragunow and Faull, 1989; VanElzakker et al., 2008). To determine whether different tau protein expression levels affect the changes in PV^+^ neuron activity mediated by A53T α-syn, we stained PV and c-fos in the SNR region of 6- and 18-month-old mice. The results indicated PV staining almost costained with c-fos, demonstrating that the remnant PV^+^ neurons were active (Figures 2A,B). It was worth noting that, accompanied by the significantly decreased PV^+^ neurons, c-fos expression was reduced accordingly in the SNR of *Pitx3-A53T*α*-Syn* × *Tau^–/–^* mice at 18-month-old (Figures 2B–D). These suggested that the number of PV^+^ neurons was proportional to the level of their activities, and *Pitx3-A53T*α*-Syn* × *Tau^–/–^* mice showed practically degeneration of PV^+^ and c-fos^+^ neurons in the SNR at old age.

### *Tau* Knockout Exacerbated Cell Apoptosis and Parvalbumin-Positive Neuron Loss Asynchronously in the Substantia Nigra Pars Reticulata of α-Syn A53T Conditional Transgenic Mice

**FIGURE 3:**
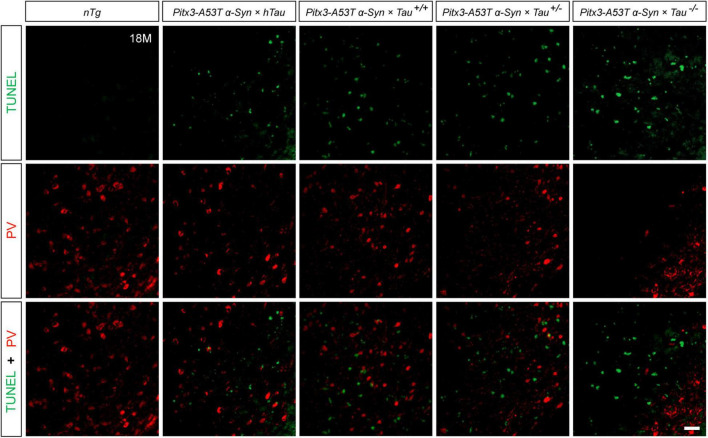
Terminal deoxynucleotidyl transferase (TdT)-mediated dUTP nick end labeling (TUNEL) (green) and PV (red) double-staining in the SNR of 18-month-old mice. Scale bar: 50 μm.

To determine whether the decrease of PV^+^ neurons was due to cell apoptosis, we stained PV and TUNEL in the SNR of 18-month-old mice. The results revealed that the less PV expressed, the more TUNEL signals were detected, as shown in the *Pitx3-A53T*α*-Syn* × *Tau^–/–^* mice. However, TUNEL did not costain with PV in the SNR of all the triple transgenic mice (Figure 3). These suggested that the degenerating PV^+^ neurons could not be labeled by TUNEL, and they might undergo a transitional stage between the expression of PV and TUNEL.

### *Pitx3-A53T*α*-Syn* × *Tau^–/–^* Mice Presented Massive NeuN^+^ Neuronal Apoptosis in the Substantia Nigra Pars Reticulata

**FIGURE 4:**
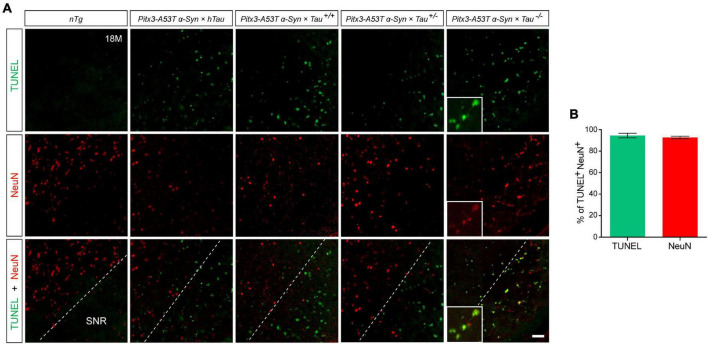
*Pitx3-A53T*α*-Syn* × *Tau^–/–^* mice presented massive NeuN^+^ neuron apoptosis in the SNR at old age. **(A)** TUNEL (green) and NeuN (red) double-staining in the SNR of 18-month-old mice. Scale bar: 50 μm. Figures in the white boxes represent the high-magnification images of TUNEL and NeuN co-staining in the SNR of 18-month-old *Pitx3-A53T*α*-Syn* × *Tau^–/–^* mice. **(B)** The percentage of TUNEL^+^NeuN^+^ co-staining cells relative to the total number of TUNEL^+^ or NeuN^+^ cells in the SNR of 18-month-old *Pitx3-A53T*α*-Syn* × *Tau^–/–^* mice. *n* = 3, values are mean ± SEM.

NeuN is a neuron-specific marker, but its expression in the SNR is species-specific. For example, the neurons in the gerbil SNR do not express NeuN, whereas the neurons in the rats’ SNR strongly express NeuN (Mullen et al., 1992; Kumar and Buckmaster, 2007; Gusel’nikova and Korzhevskiy, 2015). To determine the type of cells which presented massive apoptosis in the SNR but did not co-stain with PV, we examined TUNEL and NeuN staining in the SNR of 18-month-old mice. The results showed that TUNEL was highly costained with NeuN in the SNR of *Pitx3-A53T*α*-Syn* × *Tau^–/–^* mice (Figure 4A). The percentage of TUNEL^+^NeuN^+^ co-staining cells relative to the total number of TUNEL^+^ or NeuN^+^ cells reached 94.47 ± 2.12% or 92.72 ± 1.1%, respectively (Figure 4B). However, in the SNR of the other triple transgenic mice and *nTg* mice, the neurons rarely expressed NeuN, which did not co-stain with TUNEL either (Figure 4A). Considering the previous experimental results (Figure 3), we preliminarily speculated that NeuN may be suited for an indicator of the transitional stage of degenerating neurons in SNR between expression of PV and TUNEL, especially, at the beginning of 6-month-old.

### NeuN Gradually Replaced Parvalbumin to Mark Apoptotic Neurons in the Substantia Nigra Pars Reticulata of *Pitx3-A53T*α*-Syn* × *Tau^–/–^* Mice

**FIGURE 5:**
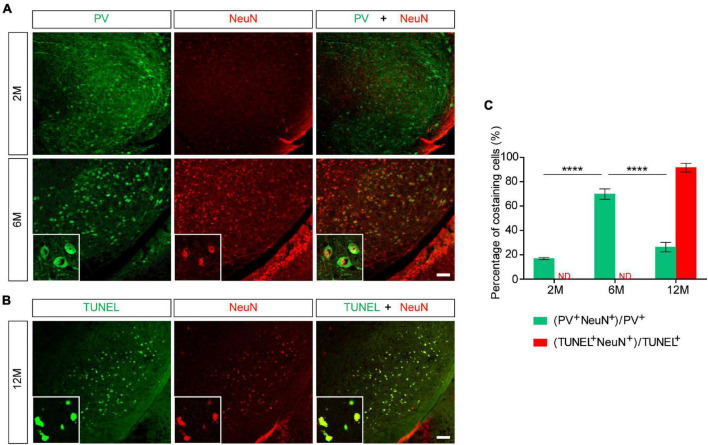
NeuN may be suited for an indicator that suggests degeneration of PV^+^ neurons in the SNR of *Pitx3-A53T*α*-Syn* × *Tau^–/–^* mice at the beginning of 6-month-old. **(A)** PV (green) and NeuN (red) double-staining in the SNR of 2- and 6-month-old *Pitx3-A53T*α*-Syn* × *Tau^–/–^* mice. Scale bar: 100 μm. Figures in the white boxes represented the high-magnification images of PV and NeuN co-staining in the SNR of 6-month-old *Pitx3-A53T*α*-Syn* × *Tau^–/–^* mice. **(B)** TUNEL (green) and NeuN (red) co-staining in the SNR of 12-month-old *Pitx3-A53T*α*-Syn* × *Tau^–/–^* mice. Figures in the white boxes represented the high-magnification images of TUNEL and NeuN co-staining. Scale bar: 100 μm. **(C)** Percentage of PV and NeuN co-staining cells to the total number of PV^+^ cells, and percentage of TUNEL and NeuN co-staining cells to the total number of TUNEL^+^ cells in the SNR of 2-, 6-, and 12-month-old *Pitx3-A53T*α*-Syn* × *Tau^–/–^* mice. *n* = 3 per genotype per time point. Values are mean ± SEM. ****P < 0.0001 (2- and 12-month-old vs. 6-month-old *Pitx3-A53T*α*-Syn* × *Tau^–/–^*). ND, not determined.

**FIGURE 6:**
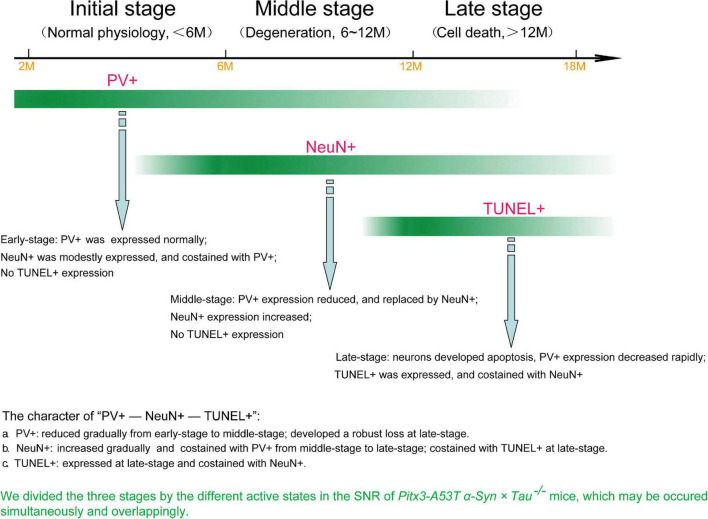
The loss of PV^+^ neurons in the SNR of *Pitx3-A53T*α*-Syn* × *Tau^–/–^* mice may undergo a transitional stage, i.e., from decreased expression of PV to increased expression of NeuN to TUNEL expression.

To further explore whether NeuN can specifically label the degenerating PV^+^ neurons, we stained PV and NeuN in the SNR of *Pitx3-A53T*α*-Syn* × *Tau^–/–^* mice at 2- and 6-months old. The results showed that PV^+^ neurons in the SNR did not begin to degenerate at 2-month-old because the expression level of PV was high while the expression level of NeuN was low (Figure 5A). At 6-month-old, NeuN was gradually increased and highly costained with PV (Figures 5A,C). Combining the previous results, while the loss of PV^+^ neurons (12- and 18-month-old) becoming significantly (Figures 2, 3), NeuN was highly costained with TUNEL (Figures 4, 5B,C). Therefore, we suspected that in the SNR of *Pitx3-A53T*α*-Syn* × *Tau^–/–^* mice, the rapidly progressed loss of PV^+^ neurons may undergo a transitional stage, i.e., from decreasing PV expression, to increasing NeuN expression, finally to TUNEL expression (Figure 6); NeuN may be a compatible marker labeling the degeneration of PV^+^ neurons at the beginning of 6-month-old.

## Discussion

In the present study, we revealed that tau knockout specifically aggravated A53T α-syn-mediated PV^+^ neurons degeneration staging and α-syn accumulation in the SNR of mice at old age (late-stage). We used the most classic TUNEL assay to stain the apoptotic neurons in the SNR and attempted to demonstrate that the neurons lost in SNR were PV^+^ neurons. Contrary to our expectations, TUNEL and PV were not co-stained. Indeed, through continuously observing the co-staining of PV and NeuN, NeuN, and TUNEL, we divided three stages by the different active states in the SNR of *Pitx3-A53T*α*-Syn* × *Tau^–/–^* mice, as shown in Figure 6. At 2–6 months (initial-stage), PV was expressed normally in the SNR, while NeuN was modestly expressed and costained with PV. At 6–12 months (Middle stage), PV expression was decreased and replaced by NeuN, which was increased obviously. At 12–18 months (late-stage), neurons developed apoptosis as TUNEL was expressed and co-stained with NeuN. So, we propose that the loss of PV^+^ neurons in the SNR of *Pitx3-A53T*α*-Syn* × *Tau^–/–^* mice may undergo a transitional stage, i.e., from decreased expression of PV to increased expression of NeuN to TUNEL expression.

Numerous studies have demonstrated that tau plays an important role in maintaining neuronal integrity and axonal transport (Augustinack et al., 2002; Combs et al., 2019). This might be because the main function of tau is responsible for the dynamic assembly of the cytoskeleton in neurons (Venkatramani and Panda, 2019). Tau depletion caused preferential loss of the labile microtubule fraction in the axon (Qiang et al., 2018). Thus, in the case of stalled growth cones of tau-depleted axons, axonal regeneration was retarded extremely (Biswas and Kalil, 2017). In the present study, we have used *A53T*α*-syn* conditionally transgenic mice to construct PD models with different expression levels of tau, all of which had developed selective loss of SNC dopaminergic neurons and severe motor coordination and balance disorders, as described previously (Jiao et al., 2020). Interestingly, we found that different degrees of SNR neuronal death were specifically induced by different tau gene dosages. At 18-mouth-old of the triple transgenic mice, PV^+^ neurons degeneration caused by tau knockout was the most significant. It indicates that tau is important for maintaining the activity of PV^+^ neurons in SNR, and tau knockout could accelerate the progression of PD mediated by A53T α-syn and promote the degeneration of SNR PV^+^ neurons.

Parvalbumin (PV) is a calcium-binding protein, which accounts for an abundant subpopulation of GABAergic neurons (Hu et al., 2014). GABAergic neurons exhibit fast-spiking patterns and form direct inhibitory synapses with the cell bodies, proximal dendrites, and starting segments of cortical pyramidal neurons (Siemian et al., 2020). Numerous studies have suggested that the number of PV^+^ GABAergic neurons in the SNR is absolutely dominant (McRitchie et al., 1996; Galaj et al., 2020). Therefore, PV can specifically mark the SNR and reflect the physiological state of this area. In this study, *Pitx3-A53T*α*-Syn* × *Tau^–/–^* mice showed specific decreased expression of PV and c-fos in the SNR at old age, which may be correlated with their increased α-syn aggregates and anxiety-like behavior. However, the mechanism of this specificity in the tau knockout state remains unclear. Uchida et al. (2014) reported that maternal stress and mutations in glutamate decarboxylase (GAD) 67, both risk factors for psychiatric disorders, can cause selective loss of PV^+^ GABAergic interneurons in the cerebral cortex. This suggests that specific degeneration of PV^+^ neurons can be associated with dysfunction of tau and GAD67, which needs further study.

Unexpectedly, our study found that tau knockout exacerbated TUNEL^+^ cell apoptosis and PV^+^ neuron loss asynchronously in the SNR of α-syn A53T conditional transgenic mice, as TUNEL did not costain with PV. This suggests that the degenerating SNR neurons might undergo a transitional stage between the expression of PV and TUNEL. Although NeuN is usually used as a definitive marker of mature neurons in neurodegenerative diseases, its role has been challenged by recent studies, indicating that NeuN staining is variable and even absent during certain diseases and specific physiological states (Duan et al., 2016). For example, NeuN expression in the SNR is species-specific. While neurons in the rats’ SNR strongly express NeuN, neurons in the gerbil SNR do not express it (Kumar and Buckmaster, 2007). Here we propose that NeuN may be an indicator of the transition of PV^+^ neurons from normal to degenerating phase in the SNR of mice. The progressive degeneration of PV^+^ neurons in the SNR of *Pitx3-A53T*α*-Syn* × *Tau^–/–^* mice may undergo a transitional process from decreased PV expression to increased NeuN expression and finally to TUNEL expression (Figure 6). Furthermore, from the results of PV, NeuN, and TUNEL staining in the SNR of the triple transgenic mice, we suppose that there may be two different types of the apoptotic neurons existing in the SNR of *Pitx3-A53T*α*-Syn* × *Tau^–/–^* mice and the other triple transgenic mice at 12- and 18-month-old: PV^+^ neurons and PV-negative (PV^–^) neurons, respectively, as classified in the previous studies (Ren et al., 1992; Fernández-Suárez et al., 2012; Mészár et al., 2012). In the SNR of *Pitx3-A53T*α*-Syn* × *Tau^–/–^* mice, PV^+^ neurons might be more susceptible to A53T α-syn-mediated cytotoxicity and underwent apoptosis.

In conclusion, our results suggested that tau knockout can exacerbate α-syn A53T-mediated PV^+^ neurons degeneration staging, from PV expression reduced to NeuN expression increased to TUNEL expression, in the SNR of mice. The rapid increase of NeuN at the middle stage and co-staining with TUNEL at the late stage made it gradually replace PV as a better indicator of the degeneration of PV^+^ neurons in the SNR. We hope that this research can provide a reference for long-term observation of the neuron degeneration in the brain of the mouse model of PD.

## Data Availability Statement

The raw data supporting the conclusions of this article will be made available by the authors, without undue reservation.

## Ethics Statement

The animal study was reviewed and approved by Institutional Animal Care and Use Committee of Sun Yat-sen University.

## Author Contributions

XL conceived the project. XL and LJ designed the experimental scheme, revised and edited the manuscript. MZ and YL conducted the major experiments, analyzed the data, and wrote the manuscript. ZX performed some experiments and aided in data analysis. All authors contributed to the article and approved the submitted version.

## Conflict of Interest

LJ is employed by Nuwacell Biotechnologies Co. The remaining authors declare that the research was conducted in the absence of any commercial or financial relationships that could be construed as a potential conflict of interest.

## Publisher’s Note

All claims expressed in this article are solely those of the authors and do not necessarily represent those of their affiliated organizations, or those of the publisher, the editors and the reviewers. Any product that may be evaluated in this article, or claim that may be made by its manufacturer, is not guaranteed or endorsed by the publisher.
